# Time in target range for body mass index and risk of new onset multimorbidity in middle-aged and older adults: a landmark analysis of two prospective cohorts

**DOI:** 10.7189/jogh.16.04134

**Published:** 2026-04-24

**Authors:** Jingru Bi, Yilin Pan, Pengcheng Ji, Wenkai Guo, Zhiwei Yin, Yuansheng Xie

**Affiliations:** 1 School of Medicine, Nankai University, Tianjin, China; 2 Department of Nephrology, State Key Laboratory of Kidney Diseases, National Clinical Research Center for Kidney Diseases, First Medical Center of Chinese PLA General Hospital, Beijing, China; 3 Department of Critical Care Medicine, Capital Medical University, Beijing Anzhen Hospital, Beijing, China; 4 Department of Nephrology, Multidisciplinary Innovation Center for Nephrology, The Second Qilu Hospital of Shandong University, Jinan, China; 5 College of Integrated Chinese and Western Medicine, Hebei Medical University, Shijiazhuang, China

## Abstract

**Background:**

Static body mass index (BMI) measurements overlook longitudinal weight dynamics. We examined the association between time in target range for BMI (TTR-BMI), which integrates stability and target attainment, and incident multimorbidity.

**Methods:**

We performed a landmark analysis using the English Longitudinal Study of Ageing and China Health and Retirement Longitudinal Study. We included participants aged ≥45 years free of multimorbidity. We calculated TTR-BMI via linear interpolation based on the trapezoidal rule to quantify continuous exposure to a target weight range. We used stratified Cox proportional hazards models to estimate hazard ratios (HRs) for incident multimorbidity. To address proportional hazards violations, we employed time-dependent coefficient analysis to examine temporal heterogeneity (≤3 years *vs.* >3 years). Lastly, we used negative binomial regression to assess secondary outcomes (disease accumulation).

**Results:**

Among 6935 participants, there were 2483 incident cases. Higher TTR-BMI was inversely associated with multimorbidity risk in a dose-response manner. In fully adjusted models, each one standard deviation increase in TTR-BMI reduced risk by 6% (HR = 0.94; 95% confidence interval (CI) = 0.90–0.99). Time-dependent analysis revealed this protection was specific to the late (>3 years) follow-up phase (HR = 0.91; *P* = 0.003), with no significant effect in the early phase. Furthermore, higher TTR-BMI was associated with a lower rate of disease accumulation (incidence rate ratio = 0.97; 95% CI = 0.95–1.00, *P* = 0.046).

**Conclusions:**

We found that TTR-BMI is an independent predictor of incident multimorbidity. Maintaining a higher TTR-BMI serves as a protective factor against disease onset. Maximising time spent in a healthy weight range offers a precise, longitudinal target for primary prevention in older adults.

Global population ageing has made multimorbidity – the coexistence of two or more chronic conditions – a major public health challenge. Multimorbidity significantly increases disability and mortality risks in older adults, leads to substantial healthcare resource use, and reduces quality of life [1]. Since established multimorbidity is generally irreversible, identifying modifiable risk factors to delay its onset is essential for healthy ageing.

Obesity, typically assessed by body mass index (BMI), is an established risk factor for chronic conditions such as cardiovascular disease and diabetes. Extensive epidemiological evidence links high baseline BMI to an increased risk of multimorbidity [2]. However, relying on a single baseline BMI measurement is limited, as it does not capture long-term changes in body weight. In older adults, weight variability often reflects metabolic dysregulation, sarcopenia, or frailty, and may be more harmful than obesity alone [3]. Therefore, novel metrics that integrate both BMI magnitude and stability are crucial for accurately predicting multimorbidity risk.

Time in target range (TTR), originally used to assess glycaemic and blood pressure control, predicts adverse outcomes better than the single time point measurements [4,5]. Recently, this concept has been applied to weight management through TTR-BMI, defined as the percentage of time BMI remains within a healthy range, reflecting both target attainment and long-term stability. However, research on the association between TTR-BMI and incident multimorbidity remains limited and is often affected by methodological flaws. Most studies used simple counting methods (*e.g.* frequency of target achievement) to define TTR, ignoring the duration and continuity of follow-up intervals [6].

To address these gaps, we analysed data from two prospective cohorts: the China Health and Retirement Longitudinal Study (CHARLS) and the English Longitudinal Study of Ageing (ELSA). We used a landmark analysis design and calculated TTR-BMI via linear interpolation to assess the impact of maintaining BMI within the target range on incident multimorbidity and disease accumulation. We hypothesised that TTR-BMI is a significant predictor of multimorbidity risk; specifically, higher TTR-BMI acts as a protective factor by maintaining metabolic homeostasis, reducing the risk of multimorbidity independent of baseline BMI.

## METHODS

### Study design and population

**Figure 1:**
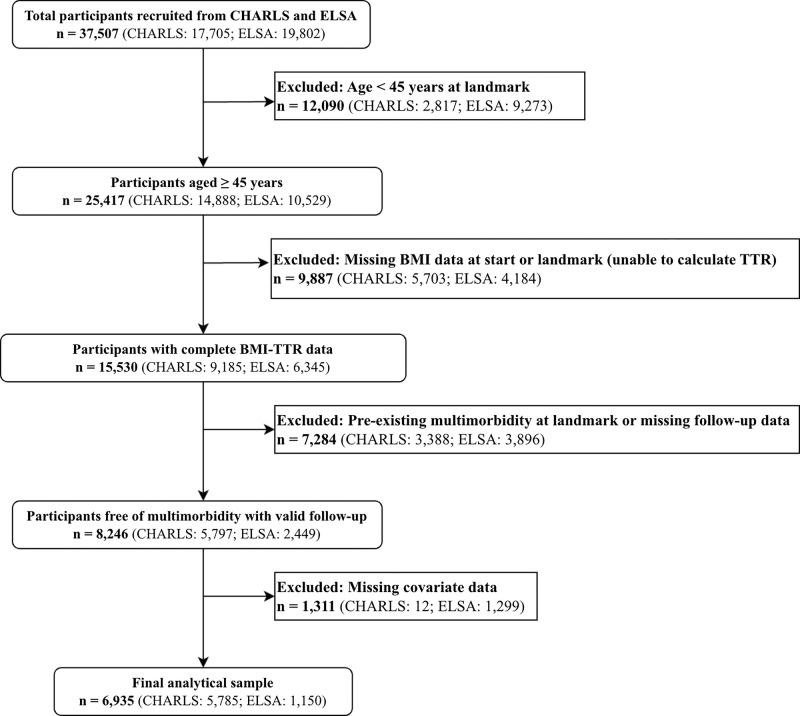
Flowchart of study participant selection. BMI – body mass index, CHARLS – China Health and Retirement Longitudinal Study, ELSA – English Longitudinal Study of Ageing, TTR – time in target range.

We conducted a secondary analysis of data from two nationally representative prospective cohorts: CHARLS and ELSA. We used a landmark analysis design to minimise bias. The exposure assessment window was 2011–2013 for CHARLS (landmark time: 2013) and 2004–2012 for ELSA (landmark time: 2012). We included participants aged ≥45 years and those with complete BMI data at both the start of the exposure window and the landmark time. We excluded participants with pre-existing multimorbidity at the landmark time or missing data on covariates or outcomes. The final analysis included 6935 participants (**Figure 1**).

### Exposure assessment: TTR for BMI

To ensure temporal clarity and comparability between cohorts, we employed a landmark analysis design. We defined the ‘exposure assessment window’ as the period before the ‘landmark time’, during which BMI fluctuations were monitored. The ‘landmark time’ (2013 for CHARLS; 2012 for ELSA) served as the anchor point, marking the end of the exposure assessment and the baseline for the subsequent follow-up period. This design aligns the study phases (exposure followed by outcome) across cohorts despite differences in calendar years.

The primary exposure was TTR-BMI during the exposure assessment window. The BMI was calculated as weight (kg) divided by height squared (m^2^). To account for ethnic differences in body composition, we used cohort-specific target ranges for normal weight: 18.5–23.9 kg/m^2^ for CHARLS (Chinese guidelines) and 18.5–24.9 kg/m^2^ for ELSA (World Health Organization standards). We calculated TTR using linear interpolation, assuming a linear change between measurements. For CHARLS, we derived TTR from 2011 and 2013 data. For ELSA (2004–2012), we maximised data utility by dividing the window into two four-year segments (2004–2008 and 2008–2012) when intermediate data were available, and averaging the TTRs of both segments. Otherwise, we based interpolation on 2004 and 2012 measurements. When BMI trajectories crossed target boundaries, a geometric method identified the exact intersection time to calculate the proportion of time within range. We expressed TTR as a continuous variable from 0 to 1, with higher values indicating longer maintenance of normal weight.

### Outcome assessment

The primary outcome was incident multimorbidity. We assessed ten common chronic non-communicable diseases: hypertension, diabetes, cancer (excluding minor skin cancers), chronic lung disease, heart disease (*e.g.* myocardial infarction), stroke, emotional/psychiatric disorders, arthritis, dyslipidemia, and asthma. Disease status was ascertained via self-reported physician diagnosis. Multimorbidity was defined as the concurrent presence of ≥2 chronic conditions. We calculated follow-up duration from the landmark time to the first occurrence of multimorbidity, death, loss to follow-up, or the end of the study (2018). Follow-up waves were 2015 and 2018 for CHARLS (maximum ~ 5 years) and 2014, 2016, and 2018 for ELSA (maximum ~ 6 years). The secondary outcome was the total count of chronic conditions at the end of follow-up, which we used to assess disease accumulation.

### Covariates

Potential confounders were collected at the landmark time, including sociodemographic variables, lifestyle factors, and baseline health status. Sociodemographic variables were age, sex, marital status (married/partnered *vs.* unmarried), and education. Due to system differences, we standardised education into a binary variable: junior high school or below *vs.* senior high school or above. Lifestyle factors included smoking and drinking status (ever *vs.* never). To control for baseline health, we adjusted for baseline BMI (continuous) and the number of baseline chronic conditions (0 or 1).

### Statistical analysis

We presented continuous variables as mean (standard deviation (SD)) or median (interquartile range), and categorical variables as frequencies (percentages). To examine dose-response relationships, we categorised TTR into low (0), intermediate (0–1), and high (1) groups. We used Kaplan-Meier curves to visualise multimorbidity-free survival, assessing differences with log-rank and trend tests.

We used Cox proportional hazards (PH) models to evaluate the association between standardised TTR-BMI (per 1-SD increase) and incident multimorbidity. Because some categorical covariates violated the PH assumption, we used stratified Cox models. In model 1, we adjusted for age and sex, stratified by cohort and baseline chronic conditions (0 or 1). In model 2, we additionally adjusted for education, smoking, and drinking, stratified by marital status. In model 3, we further adjusted for baseline BMI to isolate the effect of BMI stability.

Since the primary exposure (TTR-BMI) also violated the PH assumption, we used time-dependent coefficient analysis. We divided the follow-up into early (≤3 years) and late (>3 years) phases, with hazard ratios (HRs) calculated for each. We tested temporal heterogeneity with a‘time × TTR’ interaction term.

For the secondary outcome (accumulated chronic diseases), we used negative binomial regression to estimate incidence rate ratios, validated by Poisson regression. We stratified subgroup analyses by age, sex, and baseline disease status, testing interactions by likelihood ratio tests and Bonferroni-corrected significance levels. Lastly, we conducted three sensitivity analyses: excluding outcomes within the first two years (lag analysis), redefining multimorbidity as ≥3 diseases, and restricting follow-up to five years.

We used *R*, version 4.2.2 (R Core Team, Vienna, Austria) for all analyses. A two-sided *P* < 0.05 was considered significant.

## RESULTS

We included 6935 participants, with significant baseline differences between cohorts (**Table 1**). On average, participants from ELSA were older (69.1 *vs.* 59.6 years) and had higher educational attainment (47.1% *vs.* 10.0%) than those from CHARLS. Smoking (60.3% *vs.* 42.4%) and alcohol consumption (91.6% *vs.* 43.1%) were also more prevalent in ELSA. Additionally, median baseline BMI was markedly higher in the ELSA cohort (26.7 *vs.* 23.0 kg/m^2^). Sex distribution did not differ significantly between cohorts (*P* = 0.570).

**Table 1:** Baseline characteristics of study participants stratified by cohort*

	CHARLS (n = 5785)	ELSA (n = 1150)	*P*-value†
**Age, in years, x̄ (SD)**	59.61 (9.00)	69.10 (7.05)	<0.001
**Gender**			0.570
Male	2761 (47.7)	560 (48.7)	
Female	3024 (52.3)	590 (51.3)	
**Education level**			<0.001
Less than lower secondary	5208 (90.0)	608 (52.9)	
Secondary or above	577 (10.0)	542 (47.1)	
**Marital status**			<0.001
Married/partnered	5113 (88.4)	849 (73.8)	
Non-married	672 (11.6)	301 (26.2)	
**Smoking status**			<0.001
Never	3335 (57.6)	457 (39.7)	
Ever	2450 (42.4)	693 (60.3)	
**Drinking status**			<0.001
Never	3291 (56.9)	97 (8.4)	
Ever	2494 (43.1)	1053 (91.6)	

BMI – body mass index, CHARLS – China Health and Retirement Longitudinal Study, ELSA – English Longitudinal Study of Ageing, SD – standard deviation, x̄ – mean

*Variables are presented as numbers (percentages) unless specified otherwise.

†*P*-values are from a student’s *t* test or Mann-Whitney U test for continuous variables and a χ^2^ test for categorical variables.

**Figure 2:**
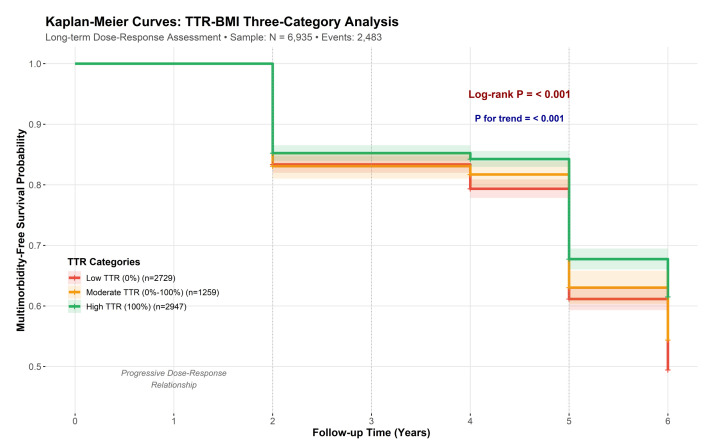
Kaplan-Meier curves: TTR-BMI three-category analysis. Shaded areas indicate 95% confidence intervals. BMI – body mass index, TTR – time in target range.

During follow-up, 2483 incident multimorbidity events occurred. Kaplan-Meier curves showed a distinct stepwise increase in multimorbidity-free survival with increasing TTR-BMI (**Figure 2**). Both the Log-rank and trend tests confirmed these significant differences and the dose-response relationship (both *P* < 0.001).

Higher TTR-BMI was consistently associated with reduced risk across all adjustment levels. In the fully adjusted model (model 3), each 1-SD increase in TTR-BMI independently reduced the risk by 5.9% (HR = 0.941; 95% confidence interval (CI) = 0.896–0.988, *P* = 0.014) (**Table 2**). Variance inflation factors were all <3, indicating no severe multicollinearity (Table S1 in the **Online Supplementary Document**).

**Table 2:** Landmark analysis of standardised TTR-BMI and risk of incident multimorbidity*

	HR (95% CI)	*P*-value
**Overall analysis**		
Model 1	0.902 (0.866–0.939)	<0.001
Model 2	0.899 (0.863–0.936)	<0.001
Model 3	0.941 (0.896–0.988)	0.014
**Time-dependent analysis**		
Early phase (≤3 years)	0.985 (0.916–1.060)	0.694
Late phase (>3 years)	0.906 (0.848–0.967)	0.003
Interaction		0.015

BMI – body mass index, CI – confidence interval, HR – hazard ratio, TTR – time in target range

*Model 1 was adjusted for age and gender, stratified by cohort and number of baseline chronic diseases (0 or 1), model 2 was adjusted for covariates in model 1 plus education level, smoking status, and drinking status, stratified by marital status, model 3 was adjusted for covariates in model 2 plus baseline BMI, time-dependent analysis was performed due to violation of proportional hazards assumption in Model 3 (*P* = 0.020), and the analysis incorporated a time-varying coefficient for TTR-BMI with a cut-off point at three years.

However, due to a violation of the PH assumption (*P* = 0.020), we performed a time-dependent coefficient analysis. The protective effect was specific to the late (>3 years) follow-up period (HR = 0.906; 95% CI = 0.848–0.967, *P* = 0.003), with no significant association observed in the early (≤3 years) phase (*P* = 0.694).

We also evaluated the impact of TTR-BMI on the accumulated number of chronic conditions (mean = 1.21; SD = 1.02). In the fully adjusted negative binomial model (model 3), TTR-BMI was inversely associated with disease accumulation (**Table 3**). Each 1-SD increase in standardised TTR-BMI was associated with a 2.7% reduction in the count of chronic conditions (incidence rate ratio = 0.973; 95% CI = 0.947–1.000, *P* = 0.046). Poisson regression yielded identical results. Furthermore, the large dispersion parameter (*i.e.* theta) in the negative binomial model indicated an absence of significant overdispersion, reinforcing the reliability of these estimates.

**Table 3:** Association between standardised TTR-BMI and the accumulation of chronic conditions at the end of follow-up*

	IRR (95% CI)	*P*-value	Reduction in count (%)
**Negative binomial regression**			
Model 1	0.945 (0.924–0.966)	<0.001	5.5
Model 2	0.944 (0.923–0.965)	<0.001	5.6
Model 3	0.973 (0.947–1.000)	0.046	2.7
**Poisson regression**			
Model 3	0.973 (0.947–1.000)	0.046	2.7

BMI – body mass index, CI – confidence interval, IRR – incidence rate ratio, TTR – time in target range

*Model 1 was adjusted for age, gender, cohort, and number of baseline chronic diseases, model 2 was adjusted for covariates in model 1 plus education level, smoking status, drinking status, and marital status, model 3 was adjusted for covariates in model 2 plus baseline BMI, negative binomial regression was used as the primary method to account for potential overdispersion in the count data, while Poisson regression was performed for sensitivity analysis.

After Bonferroni correction (*P* < 0.0056), the protective effect of TTR-BMI remained significant in participants aged <65 years and in females, with no significant interactions by age or sex. Notably, baseline chronic disease status significantly modified the effect (*P*-interaction <0.001). The risk reduction was pronounced in participants free of chronic diseases at baseline (HR = 0.899) but attenuated in those with pre-existing conditions (Table S3 in the **Online Supplementary Document**). This suggests that maintaining a normal weight confers greater preventive benefits in the disease-free state.

Sensitivity analyses confirmed the robustness of the primary findings (Table S2 in the **Online Supplementary Document**). First, after excluding participants who developed multimorbidity within the first two years to minimise reverse causality, the inverse association remained significant (HR = 0.906; 95% CI = 0.848–0.967, *P* = 0.003). Second, when multimorbidity was redefined as ≥3 chronic conditions, the association persisted (HR = 0.909; 95% CI = 0.827–0.999, *P* = 0.048). Finally, restricting follow-up to five years yielded results consistent with the primary analysis (HR = 0.945; 95% CI = 0.899–0.993, *P* = 0.026).

## DISCUSSION

To our knowledge, we are the first to evaluate the association between TTR-BMI and incident multimorbidity in older adults using landmark analysis in two large prospective cohorts. We found that higher TTR-BMI was independently associated with a reduced risk of multimorbidity and slower disease accumulation. This association persisted after adjustment for baseline BMI, sociodemographic characteristics, and lifestyle factors. Crucially, this adjustment isolated the benefit of weight stability from the magnitude of baseline BMI itself, with low variance inflation factors (<3) confirming the absence of overadjustment. Notably, time-dependent analysis indicated that the benefits of weight stability became more pronounced after three years of follow-up. Additionally, subgroup analyses highlighted that weight maintenance is particularly beneficial in the early stages of disease onset (*i.e.* in participants without baseline chronic conditions). These findings support TTR-BMI as a valuable metric for guiding long-term weight management in older populations.

Previous studies on obesity and multimorbidity have largely relied on single-point BMI measurements, overlooking dynamic weight changes. Although weight variability is linked to adverse outcomes, it is typically viewed as a negative metric without actionable clinical targets [1,2,5]. Our concept of TTR-BMI derives from the successful use of time in range in diabetes management. Unlike traditional variability metrics (*e.g.* coefficient of variation or SD), TTR-BMI integrates weight stability with guideline-recommended healthy ranges. This approach captures both consistency and target attainment. Our findings extend the utility of TTR to weight management, demonstrating that maintaining weight stability is crucial for preventing multimorbidity.

Several biological mechanisms may explain the protective effect of high TTR-BMI. First, weight instability (*i.e.* low TTR-BMI), acting as a potential risk factor, increases allostatic load through weight cycling. Repeated fluctuations exacerbate chronic inflammation, characterised by elevated C-reactive protein and interleukin-6 levels, which promote insulin resistance and cardiovascular damage [7,8]. Consistent with these mechanisms, recent evidence highlights TTR-BMI as a strong predictor of specific cardiometabolic phenotypes [9]. However, our results suggest that the benefits of weight stability extend beyond these metabolic clusters to a broader spectrum of multimorbidity, potentially reflecting preserved general physiological resilience. Second, adverse changes in body composition are critical in older adults. Weight loss often reduces lean mass, whereas subsequent regain primarily involves adipose tissue accumulation [10]. This cycle drives sarcopenic obesity, deteriorating metabolic health even with a stable BMI [8]. High TTR-BMI likely reflects preserved muscle mass and metabolic flexibility. Finally, repeated adipose tissue expansion and contraction can cause fibrosis and impaired angiogenesis. This dysfunction compromises energy buffering, leading to ectopic fat deposition in the liver, pancreas, and heart, ultimately contributing to multi-organ dysfunction [11].

Our finding that the protective effect of TTR-BMI emerged only after three years aligns with the natural history of chronic diseases. The progression from metabolic disruption to clinical organ damage is a cumulative process. Therefore, sustained weight stability is required to yield significant clinical benefits. Furthermore, the stronger association observed in participants free of baseline diseases suggests a ‘domino effect’ in multimorbidity progression. Once chronic disease is established, factors such as medication use or disease-induced cachexia may dominate health outcomes, diminishing the relative impact of weight stability. These findings underscore the value of TTR-BMI as a key strategy for primary prevention and early intervention.

The primary strength of our study is the rigorous methodological design. First, using dual-cohort data from CHARLS and ELSA validated the robustness of our findings across diverse ethnic and socioeconomic backgrounds. Second, the landmark analysis established a temporal buffer between exposure assessment and follow-up. This design minimised reverse causality and immortal time bias, addressing common limitations in previous weight-related research. Finally, calculating TTR via linear interpolation provided a more precise assessment of exposure intensity than simple counting methods.

However, our study has several limitations. First, multimorbidity was determined via self-reports. Although this method is widely accepted in large epidemiological studies, it is subject to recall bias and underreporting. Furthermore, variations in healthcare access and diagnostic practices between the two cohorts may influence disease ascertainment. However, we used cohort-stratified models to account for baseline hazard differences, and any non-differential misclassification would likely bias our results towards the null, suggesting our estimates are conservative. Second, BMI does not distinguish between fat and muscle mass. Therefore, we could not determine whether the benefits of TTR stem from stable fat mass or muscle preservation. Future studies using detailed body composition data are needed to clarify the underlying mechanisms. Third, although exposure windows differed between cohorts, we used standardised TTR proportions and cohort-stratified models to mitigate potential bias. Additionally, linear interpolation may mask short-term fluctuations between follow-up visits, potentially underestimating the impact of weight variability. Finally, despite the use of landmark analysis and extensive adjustments, causality cannot be established in this observational study, and residual confounding remains possible.

## CONCLUSIONS

We identified TTR-BMI as a robust, independent predictor of incident multimorbidity in older adults. We found that higher TTR-BMI serves as a protective factor against the onset of multimorbidity and disease accumulation, particularly during the early stages of disease onset. Incorporating TTR-BMI into routine health monitoring and prioritising weight maintenance strategies could have significant public health implications for healthy ageing.

## Additional material


Online Supplementary Document
