# Supplementary material for: Time in target range for body mass index and risk of new onset multimorbidity in middle-aged and older adults: a landmark analysis of two prospective cohorts
Source: J Glob Health. 2026 Apr 24;16:04134. doi: 10.7189/jogh.16.04134 (PMC13105787; doi:10.7189/jogh.16.04134)
Supplement: Online Supplementary Document [file jogh-16-04134-s001.pdf]

Supplement to: Bi J, Pan Y, Ji P, Guo W, Yin Z, Xie Y. Time in target range for body mass index and risk of new-onset multimorbidity in middle-aged and older adults: a landmark analysis of two prospective cohorts. J Glob Health. 2026;16:04134.

Table S1. Proportional hazards assumption test and collinearity diagnostics for the fully adjusted model (Model 3).

Variable	PH Test (*P* Value)	VIF
TTR_BMI (SD)	0.020	1.48
Age	0.685	1.07
Gender	0.364	1.92
Education	0.090	1.04
Smoking	0.576	1.77
Drinking	0.940	1.28
Baseline BMI	0.015	1.51
Global Test	0.066	—

PH – proportional hazards, VIF – variance inflation factor

The proportional hazards assumption was tested using the Schoenfeld residuals method. A *P* value < 0.05 indicates a violation of the assumption.Collinearity was assessed using VIF, with a value < 5 indicating no severe multicollinearity.Stratification variables (Cohort, Marital status, Baseline chronic diseases) were not included in the VIF calculation.

Table S2. Sensitivity analyses of the association between standardized TTR- BMI and risk of incident multimorbidity.

Analysis Scenario	HR (95% CI)	*P* Value
Primary Analysis (Model 3)	0.941 (0.896–0.988)	0.014
Lag Analysis<br>(Excluding events within first 2 years)	0.906 (0.848–0.967)	0.003
Strict Definition<br>(Outcome defined as ≥3 chronic diseases)	0.909 (0.827–0.999)	0.048
Restricted Follow-up<br>(Follow-up time limited to ≤5 years)	0.945 (0.899–0.993)	0.026

HR – hazard ratio, CI – confidence interval, BMI – body mass index, TTR – time in target range.

TTR-BMI was standardized (per 1 SD increase).All sensitivity analyses were adjusted for the same covariates as the fully adjusted model (Model 3): age, gender, education level, smoking status, drinking status, and baseline BMI, stratified by cohort and marital status (and baseline chronic diseases where applicable).Lag Analysis: Participants who developed multimorbidity within the first 2 years of follow-up were excluded to minimize reverse causality. Strict Definition: The outcome was redefined as the accumulation of at least 3 chronic diseases (instead of 2).Restricted Follow-up: Analysis was censored at 5 years to assess short-to-medium term effects.

Table S3. Subgroup analyses of the association between standardised TTR-BMI and risk of incident multimorbidity

Subgroup	HR (95% CI)	*P* Value	*P* for Interaction
Age			0.632
<65 years	0.910 (0.853–0.970)	0.004*	
≥65 years	0.945 (0.876–1.020)	0.145	
Sex			0.972
Male	0.950 (0.884–1.021)	0.164	
Female	0.909 (0.851–0.971)	0.004*	
Baseline Chronic Disease			<0.001*
0 diseases	0.899 (0.819–0.988)	0.027	
1 disease	0.954 (0.901–1.010)	0.108	

HR – hazard ratio, CI – confidence interval, BMI – body mass index, TTR – time in target range

TTR-BMI was standardised (per 1 SD increase).All models were adjusted for age, gender, education level, smoking status, drinking status, and baseline BMI, and stratified by cohort and marital status (except where the stratification variable was the subgroup variable itself).*P* values for interaction were derived from likelihood ratio tests comparing models with and without the interaction term.* Indicates statistical significance after Bonferroni correction ($P < 0.0056$, calculated as 0.05/9 tests).
